# Preclinical Models to Evaluate the Human Response to Autoantigen and Antigen-Specific Immunotherapy in Human Type 1 Diabetes

**DOI:** 10.3389/fendo.2022.883000

**Published:** 2022-04-13

**Authors:** Pamela Houeiss, Christian Boitard, Sandrine Luce

**Affiliations:** ^1^ Laboratory Immunology of Diabetes, Cochin Institute, Department Endocrinology, Metabolism and Diabetologia (EMD), Institut Nationale de la Santé et de la Recherche Médicale, Unité 1016 (INSERMU1016), Paris, France; ^2^ Medical Faculty, Paris University, Paris, France

**Keywords:** preclinical model, humanized model mouse, type I diabetes, HLA, autoantigens, antigen-specific immunotherapy, T cell assay, islet engraftment

## Abstract

Type 1 Diabetes (T1D) is an autoimmune disease that results from the destruction of pancreatic islet β-cells by auto-reactive T cells. The clinical management of T1D faces the lack of fully predictive biomarkers in its preclinical stage and of antigen-specific therapies to induce or re-induce immune tolerance to β-cell autoantigens and prevent its development. From a therapeutic standpoint, preclinical models of T1D have fallen short of directly translating into humans. To circumvent this limitation, preclinical models are being optimized to allow defining autoantigen epitopes that are presented to T cells and directly apply to the human. In this review, we propose to make a point on the latest available models such as humanized immunodeficient NOD mice models and HLA and autoantigen transgenic mice and their application in the context of T1D.

## 1 Introduction

Type 1 diabetes (T1D) is a multifactorial autoimmune disease in which T cells destroy the insulin-secreting β-cells of the pancreas. Although initially defined as a juvenile disease, it can occur at any age. It is associated on the long term with the risk of developing micro and macro-vascular complications, which makes it a major public health issue. Current diagnostic strategies in T1D patients rely on detecting anti-insulin, anti-GAD, anti-IA2 and anti-ZnT8 autoantibodies (1). However, 80 to 90% of β-cells are lost by the time of diagnosis and subjects become insulin dependent, requiring lifetime insulin delivery in the absence of therapies to revert or stop the autoimmune process responsible for the destruction of β-cells. Management of T1D remains challenging and effort should be directed towards a better understanding of the disease. Since exploring T1D in humans is difficult, the use of animal models that develop a T1D-like disease is a useful alternative. Among such models, the Non-Obese Diabetic (NOD) mouse model has been a cornerstone in studying T1D. Nevertheless, this model, alike other models in other rodent species such as the BioBreeding BB rat, fails to translate to humans in many aspects. New mouse models are required to develop screening tools and to study targeted immunotherapies in the aim to prevent or cure the disease.

## 2 The NOD Model

T1D is likely a heterogeneous disease when considering the genetic background on which it develops as well as the severity of the autoimmune process and the T cell subsets involved. The immunological characterization of T1D has been challenging considering the remoteness of the pancreas and the scarcity of autoantigen-specific T cells in the peripheral blood. For many years, the NOD mouse has allowed major advances in delineating the molecular and cellular processes of β-cells autoimmunity (2, 3). This model develops spontaneous autoimmune diabetes that shares several genetic and immunologic traits with the human disease (4). First described in 1974, the NOD mouse was used to study autoantigens, susceptibility genes, and disease initiating events as well as to characterize the nature of involved immune cells (5). It has allowed to define the successive immune steps involved in the disease process and the importance of a progressive imbalance between regulatory and effector T cells in allowing autoimmunity to proceed. NOD mice share with humans, many target autoantigens (insulin, Glutamate Decarboxylase 65, IA2/IA2b and ZnT8) and many genetic susceptibility genes (in particular, the class-II IAg^7^ gene that is homolog of the high susceptibility HLA-DQ8 class-II molecule in humans). However, NOD mice develop a considerably more extensive insulitis than in human T1D (4, 6). Also, curative strategies that were efficient in the NOD mouse have often failed to translate into a therapy to humans (7). This failure is probably related to the incapacity of this model to fully reproduce the complexity and the heterogeneity of the human disease ( (8). There are important differences between the mouse and the human both in the architecture of the islets of Langerhans and that of the immune system. Moreover, class-I and class-II major histocompatibility complex (MHC) as well as autoantigen genes, although homolog, differ in their sequence between the mouse and the human. Therefore, autoantigen epitopes that are presented by class-I and class-II HLA and H2 molecules to CD8^+^ and CD4^+^ T cells, respectively, differ between the two species (7, 9). Evidence that favors the use of antigen-specific immunotherapy to cure T1D highlights the difference in target epitopes in NOD mice as compared to T1D patients. To address these differences, many laboratories have developed preclinical humanized models to fill the gap between mice and humans and to facilitate the translation of novel discoveries to clinical trials (10, 11).

## 3 Humanized Mouse Models

Humanized mice are defined as mice engrafted with functional human cells or tissues or mice expressing human transgenes. These advanced models are designed to study the pathophysiology of T1D *in vivo*, to detect new biomarkers and to find new therapeutic targets without putting patients at risk (12).

### 3.1 HLA Transgenic Mice

Genetic susceptibility is considered a valuable clue to the molecular mechanisms of T1D. In humans, over 50 gene variants have been identified as carrying a risk for T1D, the most important being HLA genes contributing to 40-50% of the lifetime risk of T1D (13). HLA class-II genes provide the highest susceptibility. They are involved in the initiation of the T cell response to β-cells autoantigens in T1D. Among HLA class-II genes, HLA-DQ8 molecule carries the highest risk and is present in over 40% of at-risk and pediatric T1D patients (14), while HLA-DQ6 molecule is protective with a relative risk of 0.2 (15). HLA-DR molecules carry an independent risk with the particular case of HLA-DR*04:01 (14), and to a lesser extent DR*04:05 and 04:02 (16). HLA class-I molecules provide a lower risk and are associated to the progression of the disease. The strongest class-I susceptibility is conferred by HLA-B*39:06, HLA-B *57:01, HLA- B*18:01, HLA-A*02:01, HLA-A*24:02 and HLA-C*05:01 (15, 16). Class-I and class-II MHC genes directly control the peripheral T cell repertoire and the spectrum of antigen epitopes that are presented to T cells. Thus, introducing HLA transgenes in the mouse allows to characterize HLA-restricted autoantigen peptides at play in human T1D (11). “Humanized mouse” models have been developed by introducing HLA class-I or class-II transgenes into different mouse strains with or without invalidating corresponding murine H2 class-I or class-II genes (11). The expression of HLA class-I transgenes and their interaction with murine CD8^+^ T cells has been obtained using constructs encoding a human β2-microglobulin (β2m) covalently linked to HLA alpha_1_ and alpha_2_ and H2 cytosolic and transmembrane alpha_3_ chain domains (11, 17, 18). H2 class-I genes have been invalidated by deleting either the murine β2m or the MHC class-I locus. On the NOD genetic background, mice expressing HLA-A2.1 or HLA-B39 transgenes developed accelerated T1D (19–22) while the expression of HLA-DQ6 decreased the incidence of spontaneous diabetes and insulitis (23, 24). HLA-DQ8 or HLA-DR4 transgenic NOD mice depleted for IAg^7^ were resistant to diabetes probably because T cells shift toward a tolerogenic regulatory profile (25). HLA transgenic mouse models have not been limited to the NOD background. HLA transgenes have been introduced in non-diabetes prone strains, mainly C57/BL6 mice. The expression of T1D susceptibility HLA class-II genes in these mice is not sufficient to induce diabetes. However, immunizing these transgenic mice with β-cells autoantigens allowed homing of T cells to the pancreas and the development of insulitis. The expression of the human costimulatory B7.1 molecule under the control of the rat insulin gene promoter as a transgene in these mice led to the development of diabetes. HLA-DQ8/RIP-B7.1 transgenic mice showed the highest incidence, whereas the genotype HLA-DR*04:01/HLA-DQ8 attenuated this effect and HLA-DQ6/RIP-B7.1 mice were protected from diabetes (24, 26). **Table 1** shows the different HLA transgenic models that have been reported.

**Table 1:** HLA transgenic mouse models.

HLA molecules	Mouse name	Genotype	Phenotype	Applications	References
**HLA class-I**					
HLA-A*02 :01	NOD HLA-A2.1	NOD-*β2m^-/-^, HLA-A2.1/HHD^+^*	- Accelerated incidence of diabetes. -MHC class-I mediates diabetogenic immune responses.	Identification of HLA-A2.1 restricted autoantigens epitopes.	(17, 19, 20)
	NOD A2.1	NOD-*mI^-/-^, HLA-A2.1/HHD^+^*	-T1D more penetrant than β*2m^-/-^ models.* - Express murine non classical MHC class-I molecules CD1d Qa-2 and FcRn.	Test potential antibodies and serum albumin based T1D treatments.	(21)
	HLA-A^∗^02:01-transgenic (het) Ins2^KO^ NOD mice	NOD-*β2m ^-/-^, ins2^+/-^, HLA-A2.1/HHD^+^*	- Earlier onset of disease compared to *ins2^+/+^*. -Higher prevalence of diabetes in males.	- Uncover the mechanisms behind class-I VNTR alleles and T1D development - Study insulin targeted therapies.	(27)
	HLA-A^∗^02:01-transgenic Ins2^KO^ NOD mice	NOD-*β2m^-/-^, ins2^-/-^, HLA-A2.1/HHD^+^*	Increased proportion of restricted HLA-A2.1 insulin specific CD8^+^ T cells in the islets	Develop therapeutic strategies targeting insulin-specific T cells.	(28)
HLA-A*11:01	NOD HLA-A11	NOD-*mβ2m^-/-^, hβ2m^+^, HLA-A11^+^*	Reduced incidence of diabetes.	Identification of β-cell peptides in prevalent HLA class-I molecules.	(29)
HLA-B*07:02	NOD HLA-B7	NOD-*mβ2m-/-, hβ2m^+^, HLA-B7^+^*			
HLA B27	HLA B27 transgenic mouse	C57BL/6-*β2m^-/-^, hβ2m^+^, HLA-B27^+^*	Protection of diabetes.	Peptide identification for preventive therapy.	(19, 30)
HLA B*39 :06	NOD B39	NOD-*β2m^−/−^, HLA-B39^+^*	No decrease in disease susceptibility	Identification of HLA-B39 restricted epitopes.	(21)
	NOD B39	NOD-*mI^-/-^, HLA-B39^+^*	-Express murine non classical MHC class-I molecules CD1d Qa-2 and FcRn. -T1D highly penetrant. -Retain FcRn functionality.	Test potential antibodies and serum albumin based T1D treatments.	(21)
	NOD B39 with reduced thymic insulin expression	NOD-β2m^-/-^, Ins2^+/-^, HLA-B*39:06^+^	-Earlier diabetes. - Higher prevalence. - Escape of insulin-reactive HLA-B*39:06 restricted T cells from thymus.	Study the thymus escape of T cells.	(22)
**HLA class-II**					
HLA-DQ2.5	HLA-DQ2.5 KI mice	C57BL/6	-Physiological expression of HLA-DQ2.5 on immune cells in KI model compared to the transgenic model	Study autoimmune diseases especially coeliac disease	(31)
DQA1*05:01					
DQB1*02:01					
HLA-DQ8	HLA-DQ8 transgenic mice DQ8-Abo	C57BL/6- *mII^-/-^, HLA-DQ8^+^*	HLA-DQ8 restricted-GAD65 specific T cell responses after immunization. -Antibodies production. -Mild insulitis without diabetes.	Identify HLA-DQ8 restricted T cell epitopes specific of GAD65.	(32)
DQA1*03 :01					
DQB1*03 :02					
	DQ8 positive NOD mice	NOD- *I-Ag^7−/−^, HLA-DQ8^+^*	- Protection of diabetes. - hGAD65 immunization induced different GAD65 peptides than NOD mice.	Understand the role of HLA molecules along with in T1D.	(33)
	Aβ°/DQ8/NOD mice				
	HLA-DQ8 transgenic mice	C57BL/6-*IAb^-/-^, HLA-DQ8^+^*	No spontaneous diabetes.	Define the peptide restricted to HLA-DQ8.	(34)
	DQ8/mII^−^/RIP.B7-1 mice	C57BL/6- *mII^-/-^, HLA-DQ8^+^, RIP-hB 7.1^+^*	Spontaneous diabetes at 4 months age.	Study *in vivo* the diabetogenic effect of this human MHC class-II molecules.	(24, 35)
HLA-DQ6 DQA1*0103 DQB1*0601	NOD-IAb (0) HLA-DQ6 mice	NOD-*I-Ab^-/-^, HLA-DQ6^+^*	Decrease incidence glycosuria and insulitis.	Study the protective role of HLA-DQ6.	(23)
	HLA DQ6 transgenic mice	C57BL/6-*I-Ab^-/-^, HLA-DQ6^+^*	- hPPI immunization shows T cells restricted epitopes to HLA-DQ6.		(34)
	DQ6/mII^−^/RIP.B7-1 mice	C57BL/6-*mII^-/-^, HLA-DQ6^+^, RIP-hB7.1^+^*	Diabetes protection.		(24)
HLA-DR4	DRB1*0401-transgenic mice	NOD-*I-Ab^-/-^, HLA-DR4^+^*	- Do not develop diabetes. - Identified HLA-DR4 restricted T cells epitopes of human GAD65.	Evaluate the antigen-presentation capacities of the HLA-DR4 molecule.	(25, 36)
DR B1*0401 DRA1*0101					
	HLA-DRA*0101, -DRB1*0401, and hCD4 transgenic mice	BALB/c -DBA- *mII^-/-^, hCD4^+^, HLA-DR4^+^*			(37, 38)
	RIP-B7/DRB1^*^0401 mice	C57BL/6-mII^-/-^/RIP-hB7.1^+^, HLA-DR*04:01^+^	Spontaneous diabetes (25%).		(26)
HLA DR4	RIP-B7/DRB1^*^0404 mice	C57BL/6-mII^-/-^/RIP-hB7.1^+^, HLA-DR*04:04^+^,	Spontaneous diabetes (25%).		(26, 39)
DR B1*04:04 DRA1*01:01					
	HLA-DR4/GAD-TcR transgenic mice	C57BL/6-*rag2^-/-^, I-Ab^-/-,^hTCR^+^-GAD65_555–567_*	CD4^+^ T cells infiltrate in pancreatic islets with insulitis but no diabetes.	Role of hGAD65 as autoantigen in T1D.	(18)
**Complex models**					
HLA-DR3/DQ8	HLA-DR3/DQ8 transgenic mice	C57BL/6-*I-Ab^-/-^, HLA-DQ8^+^, HLA-DR3^+^, RIP-hB7.1^+^*	Spontaneous diabetes (35%).	Evaluates the modulatory effect of HLA‐DR3 on the HLA‐DQ8 restricted mice.	(35)
HLA-DQ8/DR4	DQ8DR4/RIP-B7 mice	C57BL/6-*mII ^-/-^, HLA-DQ8^+^, HLA-DR4^+^, RIP-hB7.1^+^*	Same diabetes incidence as HLA-DR4 and lower incidence than HLA-DQ8 alone.	Study regulatory role of HLA-DR4 in HLA-DQ8 positive settings.	(26)
DQA1*0301					
DQB1*0302					
and					
DRA1*0101					
DRB1*0401					
HLA DR3/DQ2 DRB1*03:01-DQA1*05:01- DQB1*02:01	HLA-DR3-DQ2 transgenic mice	C57BL/6-*mII^-/-^, hCD4^+^, HLA-DR3^+^, HLA-DQ8^+^, [RIP-hB7.1^+^]*	- Spontaneous diabetes (46%) - Similar incidence in males and females at mean age 24 weeks. - AutoAb anti-ins2.	Study role of HLA-DR3/DQ2 haplotype.	(40)
**Mouse HLA transgenic models with human autoantigens**					
HLA-A2.1-HLA-DQ8-hPPI	YES Mice	C57BL/6-DBA/CBA-*mI^-/-^, mII^-/-^, mβ2m^-/-^, ins1^-/-^, ins2^-/-^, HLA-A2.1/HHD^+^, HLA-DQ8^+^, hPPI^+^*	- Normal glucose homeostasis. - Immune cells restricted to HLA2 and HLA-DQ8 molecules and specific to hPPI in diabetic YES mice - Diabetic induction after poly I:C stimulation.	Allow the characterization of preproinsulin epitopes recognized by CD8^+^ and CD4^+^ T cells and specific to human insulin autoantigen.	(41)
HLA-A2.1-HLA-DQ8-hPPI-hB7.1	YES-RIP-hB7.1	C57BL/6-DBA/CBA-*mI^-/-^, mII^-/-^, mβ2m^-/-^, ins1^-/-^, ins2^-/-^, HLA-A2.1/HHD^+^, HLA-DQ8^+^, hPPI^+^ [RIP-hB7.1^+^]*	- Spontaneous diabetes in males and females - Immune cells restricted to HLA2 and HLA-DQ8 molecules and specific to hPPI and spliced hPPI in diabetic YES-RIP-hB7.1 mice.	-Evaluate the relevance of Tcell assays in the diagnosis of T1D. -study of modified hPPI peptides - Evaluate peptide immunotherapy that would directly apply to human diabetes. - study the mechanisms triggering T1D.	(42)
Human insulin	HuPI mouse	NOD/Lt-*Ins1em1(INS)Tkay*	-Normal glucose homeostasis -Lower incidence of diabetes then NOD mice - Delayed insulitis	-Assess the role of insulin in T1D -Highlight the importance of CRISPR/Cas9 in humanized models	(43)
HLA-DQ8-GAD65	DQ8 and RIP7-GAD65 double transgenic mice	C57BL/6-*mII^-/-^, HLA-DQ8^+^, hGAD65^+^, RIP-hB7.1^+^*	Insulitis after immunization with GAD cDNA.	Test susceptibility genes of diabetes.	(44)
	Double transgenic (DQ8-GAD65) mice	BTBR- *mII^-/-^, HLA-DQ8^+^, hGAD65^+^, RIP-hB7.1^+^*	Immunization by GAD antigen specific insulitis develop diabetes.	Study role of human GAD in diabetes.	(45)

HLA transgenic mice allowed the identification of key players in T1D development. Adoptive transfer of T cells from HLA-DQ8 transgenic mice immunized with GAD65 and having evidence of insulitis, induced insulitis in recipients (32). Challenging HLA-DR4/RIP-B7.1 mice with murine proinsulin-2 peptides accelerated T1D development (46). The level of thymic *insulin 2* gene expression determined the timing and the incidence of T1D in an HLA-B*39:06 transgenic mouse, a similar effect of that of the invalidation of the *insulin 2* gene in NOD mice (47) or of the insulin variable number of tandem repeats (VNTR)in humans (22).

### 3.2 HLA and Human Autoantigen Transgenic Mice

Besides pointing to the role of HLA in T1D development, HLA transgenic models have allowed identifying β-cell peptides recognized by T cells using either T cell hybridomas, or T cell assays or class-I or class-II peptide-MHC tetramers (29, 34, 38, 48). The main autoantigens that are recognized by T cells in T1D patients are insulin and its precursor preproinsulin (PPI) hereafter described as insulin, GAD65, ZnT8, IA2 and islet-glucose-6-phosphatase catalytic subunit-related protein (IGRP) (49, 50). Differences in β-cell peptides have been seen depending on whether a murine or a human autoantigen was expressed (34). Therefore, HLA transgenic mice that express human GAD65 or human PPI have been developed (41, 44).

#### 3.2.1 Humanized Mice That Express Human Insulin

PPI is synthetized in β-cells and translocated to Endoplasmic reticulum (ER) in the form of proinsulin after cleavage of signal peptide sequence by a peptidase. Proinsulin is later converted into mature and bioactive insulin (51). Among the autoantigens, insulin has been ascribed a key role in T1D (52, 53). In infants followed from birth, anti-insulin antibodies were detected early in the diabetes process in at risk subjects (54). The genetic polymorphism of a VNTR 5’ of the *INS* gene confers a significant risk for T1D development (55). PPI epitopes that are presented by different HLA class-I molecules to CD8^+^ T cells and by HLA class-II molecules to CD4^+^ T cells have been characterized in patients and in mouse models (50).

In mice as well as in some fish species, two genes located on different chromosomes encode respectively insulin 1 and insulin 2 (56). However, humans carry a unique *Insulin* gene that shows homology with the murine *Insulin 2* gene. Murine insulin 1 and insulin 2 differ by two amino acids located in the insulin B chain at positions B9 and B29 and by amino acids located in the insulin leader and C-peptide sequences. The *Insulin 1* gene lacks an intron that is present in *Insulin 2* and in the human *Insulin* gene. In the mouse, insulin 1 is the main insulin isoform secreted in the pancreas whereas insulin 2 predominates in the thymus. Normal glycemia was maintained in the absence of either *insulin1* or *insulin2* on conventional mouse genetic backgrounds (43, 57). However, the invalidation of the *Insulin 1* or the *Insulin 2* gene led respectively to prevent and accelerate T1D development in the NOD mouse (47, 58) while invalidation of GAD or IA2 gene had limited effects. In genetically modified mice, human transgenes are randomly integrated in the genome which may lead to abnormal gene expressions and functions (59). This raises concerns about losing the insulin physiologic function when replacing murine insulin with human insulin. Nevertheless, using a PPI transgene in NOD models invalidated to *Insulin 1* and *Insulin 2* genes restored the metabolic function of insulin even when switching tyrosine to alanine at position B16 (57). Also, YES mouse that lacks the expression of murine MHC class-I, class-II and insulin genes and expresses human insulin (hPPI), HLA-A*02:01 and HLA-DQ8 transgenes, showed normal β-cell mass and normal glycemia values even after intraperitoneal injection of glucose (41).

#### 3.2.2 Implications of HLA Transgenic Mice Expressing Human Autoantigens

HLA transgenic mice modified to express human autoantigens allow mapping T cell epitopes that match human epitopes, especially in case of autoantigens with low expression in mice (11). These mice highlight the importance of certain antigens in the initiation of diabetes and allow the detection of specific T cells in the pancreas. Immunization with hGAD cDNA induced insulitis and glucose intolerance in HLA-DQ8/mII-/RIPB7.1-hGAD65 transgenic mice (36). When immunized against hPPI, YES mice showed insulin specific T cell responses that are restricted to HLA-A*A2:01 and HLA-DQ8 molecules (41). These mice developed diabetes when injected with polyI:C (Toll-like receptor 3 agonist) and spontaneous diabetes when co-expressing RIP-hB7.1 along with CD8^+^ and CD4^+^ T cell responses that largely overlap (41, 42). Thus, in addition to refining the study of human susceptibility genes and human autoantigen epitopes that are targeted by T cells in T1D, humanized models allow evaluating the role of environmental factors in triggering T1D development.

### 3.3 Humanized Immunodeficient NOD Models

**Figure 1:**
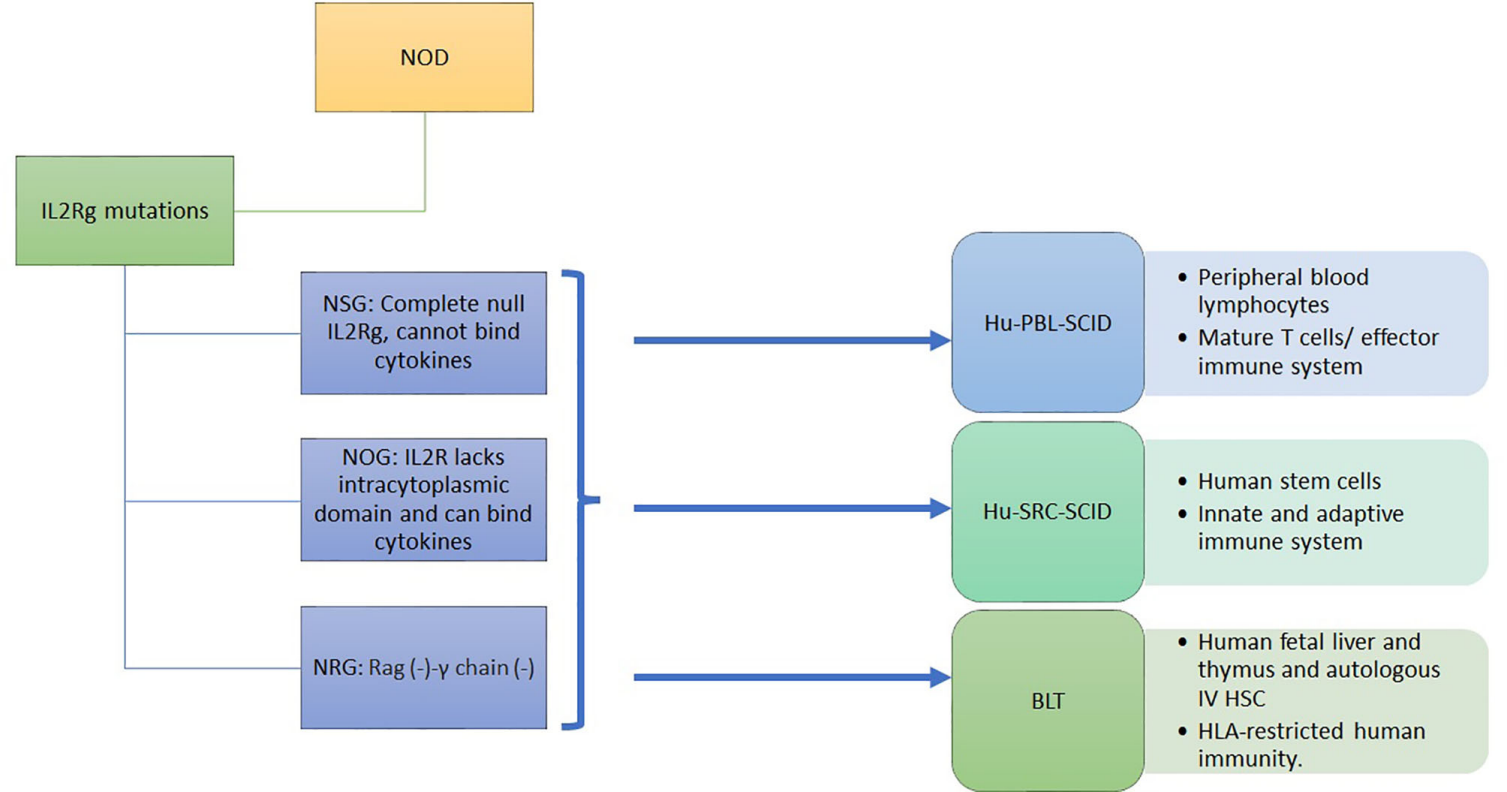
Immunodeficient humanized mice model with functional human immune system.

HLA transgenic NOD models has been a unique model to advance our understanding of T1D. However, the genomic inflammatory responses in humans and mice do not overlap, possibly explaining the failure of translating therapies from mice to humans (60). Immunodeficient mice engrafted with human immune cells and tissues provide NOD mice with a humanized functional immune system to overcome this problem. Immunodeficient NOD (NSG) mice were obtained by deleting the *IL2-receptor γ C* gene, although not the *SCID-Prkdc* gene, from NOD-SCID mice (61). These mice are engrafted by a human immune system and/or by human islets (8). In NSG mice, the lack of B, T, and NK cells and the poor lymph node organization and development support the engraftment with human cells and tissues. The human immune system engraftment could originate from human peripheral blood monocytes or from human stem cells isolated from the umbilical cord, from fetal liver or mobilized to the periphery through G-CSF. It can also be obtained by transplanting the human fetal liver and autologous thymus fragments under the renal capsule while injecting the autologous human HSC intravenously (8, 12) (**Figure 1**). This leads to murine models harboring a functional human immune system. The proper technological approach for engraftment and the proper mouse model are chosen depending on study objectives, i.e study of autoreactive or alloreactive T cells, or HLA-restricted epitopes, or induction of autoimmune diabetes. However, the scope of these models is limited by the murine component of many immune determinants: cytokines, murine major histocompatibility complex (H2), homing molecules, poorly developed lymph nodes and in case of diabetes, the cutoff level of a normal glycemia (62, 63).

To optimize these humanized models, mice can be manipulated to induce diabetes or to express human genes such as IL3, MG-CSF, SCF, thrombopoietin, SIRP alpha and HLA class-I or class-II depending on the study outcome (62, 64). **Table 2** shows the hyperglycemic and the HLA transgenic NSG mouse models.

**Table 2:** HLA Transgenic immunodeficient mouse models.

Mouse model	Genotype	Properties	Advantages	References
**Diabetic models to study the function of human islets and stem cell–derived β-cells in the absence or presence of an alloreactive human immune system.**				
NSG-STZ	NOD/Lt-*scid-Il2rγ-/-*	- Chemically induced diabetes (STZ). -Islet engraftment reverse hyperglycemia. - HLA-mismatched human PBMC return hyperglycemia.	Hyperglycemia induced at will, engraftment with functional human system.	(65)
			Human islet allograft rejection model.	
NRG akita	NOD-*rag1^-/-^, Il2rγ^-/-^, ins2^akita^*	- Monogenic model of diabetes, not auto-immune. - Spontaneous diabetes. - Normoglycemia if human islet transplantation.	Study hyperglycemia effect on beta cell proliferation.	(66, 67)
NSG RIP-DTR mouse	NOD-*scid*-*Il2rγ^-/-^, RIP-DTR^+^*	Administration of diphtheria toxin leads to beta cell destruction and hyperglycemia	Control timing of induction of hyperglycemia.	(68, 69)
			No toxicity.	
			Homogeneity.	
			Irreversible hyperglycemia	
**HLA transgenic models to study the human autoreactive immune cells *in vivo* using PBMCs or HSC engraftments from T1D patients**				
NSG-A2 mice	NOD-*scid-ragγc^-/-^, HLA-A2^+^*	- Detection of transduced HLA-A2.1 restricted CD8^+^ T cells expressing TCR specific for 3 IGRP epitopes in the blood, spleen and pancreas up to 5 weeks post-transfer - *In vitro* reaction to specific peptides	Evaluation of T cell‐modulatory interventions in an *in‐vivo* system.	(70, 71)
		- Transfer of PBMC from HLA matched T1D patients and healthy donors. - The immune system from T1D donors had a higher capacity to infiltrate the pancreas and produce insulitis. - IGRP, IAPP, Insulin, IA2 specific CD8^+^ T cells were detected.	Identify HLA A2 restricted epitopes recognized by CD8^+^ T cells.	(17, 72)
HLA-DQ8–Tg Hu-Mice	NOD-*scid-ragγc^-/-^, HLA-DQ8^+^*	- Engrafted with HLA-DQ8 human fetal thymus and CD34^+^ fetal liver cells into HLA-DQ8 transgenic mice. - Develop diabetes after low dose STZ injections and autologous HLA-DQ8 insB_9-23_ TCR transfer or insB_9-23_ immunization. - Insulitis.	Study pathogenesis of T1D and the role of insulin in inducing T1D.	(73)
			Test for therapeutic interventions.	
NSG.DR4 mice	NOD-*scid-ragγc^-/-^, HLA-DR4^+^*	- Auto antigen expanded HLA-DR4 restricted CD4^+^ T cells from T1D patients induce insulitis with reduction in insulin expression and increased beta injury.	Understand mechanisms of induction of human diabetes.	(74)
TCR.DR4 mice	Tcrb−/−, I–Ag7+/+, DR4Tg/0	-Lack α/β T cells, and express the human DR4 transgene - Models with TCR that retains the binding specificity of the human TCR but can interact with the mouse CD3 signaling complex	Carry multiple different TCRs, both autoreactive and control TCRs.	(75)
			Study the peptide- HLA based therapies	
DR4Tg mice	NOD.HLA-DR4Tg.H2Ab1-/-.Rag1-/-	-Express HLA-DR4 transgene -Retrogenic mouse model expressing TCR reative to GAD65_115-126_ native or deamidated peptides - GAD65 specific T cells infiltrates the pancreas after immunization	- Study the immunogenicity of GAD65 peptides -Study the phenotype of activated CD4^+^ T cells -Study the response of T cells against PTM epitopes in T1D and their role in inducing autoimmune diabetes.	(76)
NSG Ab0 –DR4 mice	NOD.Cg‐*Prkdc^scid^, Il2rg^tm1Wjl^ H2-Ab1^tm1Gru^, HLA‐DRB1^+^*	Transfer of GAD TCR‐transduced primary human CD4^+^ T cells HLA DR4 restricted causes insulitis without overt diabetes.	Study of human T‐cell modifications *in vivo*, development of human disease models that incorporate human T cells.	(77)

These models are valuable to decipher the pathophysiology of T1D. They serve to study T1D triggering factors. Fifty percent of NSG mice transplanted with human islets and infected with cocksackie virus developed hyperglycemia (78). Also, these models serve in studying human β-cells proliferation *in vivo*. NSG strains have been genetically modified to develop hyperglycemia either spontaneously or chemically (79). These hyperglycemic models could be transplanted with human islets or human stem cells derived from β-cells or progenitor cells to revert the hyperglycemia (66). Engraftment of hyperglycemic NRG Akita mice with human islet cells increased the β-cell proliferation by 6 folds as compared to normoglycemic NRG Akita mice (80).

Additionally, such models allow to identify key players in T1D and mechanisms behind β-cells destruction. Destruction of pancreatic islets and infiltrates with human CD4^+^ T cells was observed in humanized NSG mice after injection of irradiated monocytes from diabetic NOD mice (81). The adoptive transfer of T cells transduced to express human autoantigen-specific TCR allows to isolate a larger number of human diabetogenic T cells and dissect the role of islet autoantigens in T1D. A mouse model can be engrafted by human or murine stem cells transduced to express autoantigen-specific TCRs to create retrogenic humanized models. The retrogenic mouse model allows to study human autoreactive T cells phenotype and function and to study thymic selection (82). The retrogenic mouse can express multiple autoreactive or control TCRs to better mimic the physiological setting. In a TCR-transgenic humanized mouse model, thymocytes expressing TCRs specific to the HLA-DQ8 restricted peptide hPPI 33-47 (insulin B9-23) were negatively selected in an HLA-DQ8 positive human immune system more efficiently than on an HLA-DQ8 negative immune system (83). In another model, HLA-DR4 retrogenic mouse expressing monoclonal or polyclonal TCRs reactive to native or deamidated GAD-65_115-127_ peptides showed that post-translational modifications epitopes do not support T reg development (76).

### 3.4 Applications of Humanized Models in T1D Diagnosis, Treatment and Prevention

The diagnosis of autoimmunity in full-blown T1D is based on the detection of autoantibodies (1). However, in prediabetic individuals, while positivity for three or four different antigenic specificities is highly predictive, positivity for one or two autoantibodies has a low predictive value, highlighting the importance of developing new assays for early and accurate diagnosis (84). Providing the key role of T cells in driving the autoimmune process against β-cells, T cell assays need to be developed. T cell responses to epitopes recognized in the context of HLA class-I and class-II transgenic mice will be useful in helping to develop these assays for diagnosing and immune monitoring in patients under immunotherapy.

Identifying epitopes recognized by T cells in T1D pave the way to developing antigen-specific immunotherapies which are likely to carry a high benefit/risk ratio (85). Among recent examples, injecting NOD-β2m^null^ HHD mice with a nanoparticle-peptide complex (PSB coupled to HLA-A2 restricted ZnT8 or IGRP epitopes) induced immune tolerance and prevented diabetes by decreasing the numbers of autoreactive CD8^+^ T cells (86). Altered peptide ligand for insulin B1(5-14) induced antigen-specific anergy in a similar model (87). Vaccinating NSG-HLA-DQ8 transgenic mice with insulin mimotopes stimulated Foxp3^+^ Tregs *in vivo* (88).

These models can allow discovering and testing new-targeted therapies. The study of teplizumab in HLA-A2/NSG mice allowed the identification of CCR6^+^ Treg cells secreting IL-10 which could be considered as a therapeutic target (89). HLA-DQ8/hGAD65 transgenic mice have been used to test a targeted therapy using GC7 molecule which inhibits the eucaryotic translation initiation factor A-1 (eIF5A) activating enzyme. In this model, the onset of T1D was delayed and the function of β-cells improved (90).

In other preclinical models, humanized mice engrafted with human immune precursors have been used to evaluate the translational potential of promising therapies. Currently, pancreatic islet transplantation can restore normoglycemia in patients with long-onset T1D. However, it faces the shortage in human donors and the risk of graft rejection. Manipulation of the hematopoietic stem-cells or PBMC engrafted NSG strain has generated mice in which chemically or spontaneously induced diabetes was reversible by islet engraftment (61, 91). This allowed the identification of new potential therapeutic targets and the study of the mechanisms of islet graft rejection and the means to prevent this rejection (92). Combining human immune system and islet engraftment in these models allow the optimization of protocols for inducing remission in T1D through islet engraftment and suppression of graft rejection. Treatment with IL-2 and rapamycin suppressed effector T cells and stimulated regulatory (CD4^+^FOXP3^+^) T cells reducing human islet allograft rejection in NSG mice transfused with human spleen mononuclear cells (93). Combination therapy with ethylcarbodiimide, rituximab and rapamycin limited the rejection of xenogeneic porcine islets in humanized mice (94).

As another approach, costimulation blockade has been shown to prevent the rejection of allogeneic pancreatic endoderm by human PBMCs in a humanized model *in vivo* (95). Co-transplantation of human bone marrow-derived mesenchymal stem cells (hBMSCs) could prevent immune rejection and improve human islet transplantation in a humanized NSG mouse (96). Another NSG mouse model was created by transferring genetically modified human embryonic stem cells that lacked CIITA and expressed HLA-A2 as the only HLA class-I molecule. The differentiation of these cells into β-cells then the engraftment with human PBMCs allowed to study the immune response and the islet rejection (97). Genetically modified β-cells engraftment is another promising therapy to prevent T1D recurrence post engraftment; human β-cells engineered to express Herpesvirus encoded immune-evasion proteins prevent islet destruction in NSG mice by degrading MHC class-I molecules and inhibiting granzyme B activity (98). Beyond allograft rejection, NSG mice can be used to study xenogeneic GVHD reactions. An option has been developed that replaced human islets by genetically modified porcine islet. Engraftment of neonatal porcine islet-like cell clusters overexpressing CTLA-4 Ig analogue in diabetic Hu-HSC-NSG mice reverted diabetes without a xenogeneic GVHD reaction (99). Despite these advantages, the translation of treatment to humans is not straightforward. The dosing, frequency, and route of administration of immunotherapies are still to be refined.

### 3.5 Other Humanized Models for T1D

T1D involves an auto-immune destruction of the β-cells. Therefore, a therapeutic approach aiming at modulating the immune response represents an attractive means of treatment approach. So far, therapies have met with varying clinical success despite efficiency in murine preclinical models. At best, the response to short-term treatments such as anti-CD3 antibodies had time limited effect. Pre-clinical models expressing the human targeted molecules might fill this gap and allow the optimization of therapeutic protocols. Immunomodulatory treatments have been attempted. Humanized murine models expressing human CD3ϵ and CD20 were developed to study the therapeutic potential of combined protocols in restoring tolerance in T1D (100). Treating VH125.hCD20/NOD mice with anti-human CD20 delayed diabetes development by reducing the effect of costimulatory molecules on B cells, by decreasing the INFγ production and by limiting T cell activation in the islets. Combining a histone deacetylase inhibitor with low-dose CD3 antibodies abrogated local inflammation, improved pancreatic β-cell survival and metabolic function, and led to long-lasting diabetes remission (101).

β-cell antigen-based therapy is another attractive approach, as it precludes the long-term side effects of immune modulating therapy as being antigen-specific. To study the capacity of dendritic cells to induce an antigen-specific immune tolerance, a humanized mouse model expressing human CD205 on a NOD background was produced. CD205, as the endocytic receptor of antibodies coupled to islet antigens on myeloid dendritic cells, allows antigen processing and presentation by MHC class-I and II and modulate antigen-specific T cell responses (102).

## 4 Perspective

Humanized HLA and autoantigen transgenic mice allow the identification of the epitopes restricted to HLA class-I and class-II molecules paving the way to antigen-specific immunotherapy and to restoration of immune tolerance in T1D patients. Effort should be made to regenerate β-cell mass after reestablishment of tolerance to autoantigens in T1D patients with a low β-cells mass (103). Using humanized immunodeficient models engrafted with human immune system and human β-cells in the context of human susceptibility genes will allow a better understanding of the pathophysiology. Replacing the current engraftment techniques with the induced pluripotent stem cells (iPS) technology might provide better means to study the disease. These cells can be isolated from T1D patients and can differentiate into β-cells, hematopoietic stem-progenitor and thymic epithelium (61, 104). These models also allow to identify new biomarkers and to design new screening and prognostic biological assays that can apply to humans. This personalized *in vivo* model provides new insights into the immune function of patients with T1D. This allows to have a better understanding of diabetes in the individual and to overcome the heterogeneity of the disease. It will facilitate the development of peptide-based predictive, diagnostic, and therapeutic strategies and will pave the way to personalized medicine.

## Author Contributions

All authors contributed to the article and approved the submitted version. PH designed, did the literature review, and wrote the article. SL designed, reviewed, and edited the review. CB designed, reviewed, and edited the review.

## Funding

The publishing of this review is funded by Association Robert Debré pour la recherche médicale (ARDRM). This work was supported by « Fondation Servier » and « Fondation pour la Recherche Médicale » (FRM).

## Conflict of Interest

The authors declare that the research was conducted in the absence of any commercial or financial relationships that could be construed as a potential conflict of interest.

## Publisher’s Note

All claims expressed in this article are solely those of the authors and do not necessarily represent those of their affiliated organizations, or those of the publisher, the editors and the reviewers. Any product that may be evaluated in this article, or claim that may be made by its manufacturer, is not guaranteed or endorsed by the publisher.
